# Observations on Fractures of the Patella

**Published:** 1828-08

**Authors:** Charles Bell


### Art. III. *Observations on Fractures of the Patella.* By Charles Bell, F. R. S. *As delivered by him in his Clinical Lectures.*

[From the London Medical Gazette.]

Gentlemen,—The cases to which I shall first direct your attention, are instances of fracture of the patella, now in the hospital; and although they are related briefly, they contain illustrations of important practical doctrines; indeed, they guide us to the principles of our treatment in all cases of fracture.

*First case.* Mary Langthrone, aged 46, was admitted into the females' accident ward, August 23d. Whilst she was at work in the kitchen, she heard her mistress call her, and as she was running up stairs, her foot slipped from one of the steps, and she fell. She is not certain whether her knee struck against the step. She was unable to raise herself up, and when her master lifted her, she discovered that her lameness was occasioned by her kneepan being broken. When brought to the hospital, there was no appearance, on the integuments of the part, as if it had been struck or bruised. The patella was fractured transversely, and tumefaction of the joint arose soon after her admission. During the first week, attention was paid to the position of the limb and body. She was propped up in bed to bring forward the origin of the rectus muscle; the limb was extended to relax the ligament of the patella; and cold lotions were applied. When the knee was in a state to admit of the fracture being set permanently, it was done in the following way:—The limb was in the first place rolled from the ancle to the middle of the thigh; compresses were then placed above the upper portion of the patella, and below the lower portion; straps, made of portions of bandage, were laid along each side of the knee, one on the inside and another on the outside. A second bandage was now applied over the compresses and the straps; it was passed over the central parts only of the latter, and the ends were left loose. These extremities of the longitudinal straps were tied closely together on each side of the knee-joint: in this manner the folds of the roller and the compresses were approximated towards the centre of the knee; and the two fractured portions of the patella were thus brought close together.

Oct. 20.—It is now about eight weeks since the accident, and the bone is united, but, it is feared, by ligament, although there is scarcely any perceptible distance intervening between the two portions. She has attempted to walk about the ward, but finds the limb very weak, and being timid, she seldom ventures to rise from her bed.

*Second case.* David Keith, aged 36, a bricklayer, was brought to the hospital, Sept. 15th, in a state of intoxication, having received an injury on the knee. Little credit can be given to the account which he himself afterwards gave of the manner in which the accident occurred; but it appears from the statement of those who were with him, that his foot slipped upon the curb-stone, so that he fell with his whole weight on his left knee, which struck upon the iron grating of a sewer. When brought to the hospital, there was great ecchymosis over the whole front of the knee-joint; and the patella was discovered to be broken into three portions. There appeared to be a transverse fracture separating two upper and lower pieces, while a lateral portion was broken off longitudinally, and was found lodged on the inside of the condyle of the femur. This last-mentioned portion was replaced in its proper situation close to the other two. He was placed in a sitting position in bed, with orders to keep his limb constantly in a state of extension. When he became fatigued with sitting erect, he was allowed to recline backwards, but care was taken to elevate the limb upon pillows, so that the knee approached the pelvis, and the rectus muscle was relaxed. Seventeen leeches were applied to the joint, and afterwards cold spirituous lotions were kept constantly upon the parts, until the active inflammation was subdued. A bandage was applied to the limb, but as it gave pain, it was immediately taken off. When the inflammation had ceased, the knee was bandaged.

October 25th.—The union is so complete, that there is great difficulty in distinguishing where the bone was fractured. The skin which covers the patella being no longer thickened or condensed, but in its natural condition, the whole surface of the patella is found to be uniform; no indentation can be detected, to mark any deficiency of solid bony union: there is a slightly elevated ridge of bone extending transversely, which points out the situation of one of the fractures. He has been walking about the ward for some days, and is to leave the hospital to-morrow.

These two cases serve to illustrate a distinction in the kinds of fracture of the patella, which it is of the greatest consequence the practitioner should understand. In the first it was not clearly made out, from the statement of the patient, how the accident took place. For it may be a question whether the patella was fractured by the convulsive action of the muscles while she was endeavoring to save herself from falling, or whether on falling she struck the patella against the edge of one of the steps, and thus fractured it, independently of the action of the muscles. It appears, however, that the first supposition is most probable: since there was no mark of any bruise upon the integuments of the knee, and there was very inconsiderable swelling of the joint consequent on the injury.

In the second case, even although we had not the statement of the person who saw the accident, there was evidence of great injury having been inflicted. The bone was fractured into three portions. This proved that it was not by the simple tearing and snapping of the bone by the action of the muscles, that the accident occurred. Besides, there was great ecchymosis, with swelling and heat of the joint for some time. It appears, then, that the patella in the last instance was fractured by the patient's tripping upon the edge of the curb-stone, and falling upon the knee with his whole weight against the iron spokes of the grating. This accident, besides fracturing the patella, it is to be observed, produced great injury to the surrounding structures.

We shall consider these cases separately, for it is important that we should understand clearly the distinctions between them. In the first place, we shall attend to that fracture of the patella, which is occasioned by the convulsive action of the extensor muscles of the limb, in which little injury is done to the parts around the joint: and then we shall contrast it with another kind of fracture of the patella produced by a direct blow upon the bone, by which it is splintered against the heads of the femur and tibia; as, for example, when a person receives a kick from a horse, or falls from a great height upon his knee. I remember it happening thus:—A coachman in the act of pulling up his horses, broke the foot-board on which he pressed with his whole weight, and fell from his box. In his fall he struck his knee against the roller bolt, and fractured the patella into several portions. In such a case there is not only a fracture of the patella, but there is also a violent injury of the joint combined with it, and the importance of the distinction is evident from this,—the treatment, which if pursued in the one case may be perfectly proper and successful, if followed in the other may be attended with the loss of life. But there is, besides, another reason; there is a prospect in the one case of the broken portions being united, merely by means of ligament, while we may expect in the other, by proper management, to procure union by bone.

In the common fracture of the patella, we observe an appearance of unnatural flatness, and squareness of the fore part of the knee, which is very peculiar and characteristic of this injury; when once it has been observed, the appearance can scarcely in future be mistaken. This squareness is occasioned by there being certain points of bone brought nearly upon the same level; the patella being transversely divided, its upper portion is drawn away from the lower, and thus the usual prominence in the centre of the knee is destroyed; the fractured portions, now occupying the upper and lower parts of the joint, appear as projections there, while the two condyles of the femur jut out on each side unnaturally, and a square flat surface is produced, bounded by these projections of bone. In placing the hand upon the knee, the four knobs of bone which constitute this peculiar squareness can be felt, and the sensation conveyed makes it quite clear what the nature of the accident is.

This kind of fracture of the patella is most frequently caused by the person stumbling, and then making a violent effort to recover his balance. He suddenly brings the quadriceps extensor into powerful exertion, and the force of this muscle may be imagined, when we consider that it is capable of throwing up the whole body as in a leap: it is this muscle too which straightens and then fixes the limb, when we rise up under a heavy load placed upon the shoulders. We can, therefore, imagine, that in making a violent convulsive effort, when the body is thrown off its centre of gravity, this muscle may act with force sufficient to rend the patella asunder. But there is another circumstance which renders the liability to fracture greater; there is a certain degree of flexion of the knee-joint, during which the patella is more easily broken. About the middle state between extreme flexion and extension of the limb, the patella rests with its upper half upon the convex surface of the femur, while that part of the bone to which the ligament of the patella is attached, projects without support over the space left between the separating surfaces of the femur and tibia. If the knee-joint be further bent, and the quadriceps muscle be in a state of spasmodic contraction, thus retaining the patella fixed upon the end of the femur,—and if the ligament of the patella be acting in an angular direction upon the projecting part, the bone is then snapped, as it were a stick broken across the knee.

Sometimes in cases of fracture of the patella, which take place in this way, we find there is a confusion in the statement of the patient. When asked how it happened, he is at a loss to make out whether the bone was broken before he fell to the ground, or whether his fall caused the fracture. But it is not unfrequent that intelligent patients, of their own accord, state distinctly that they heard a snap at the knee-joint when they were making a violent exertion, and immediately after that they found they were lame, the knee sinking under them. It is a remarkable circumstance attending this accident, that when a patient has once suffered from it, he is very liable to have the patella of the other knee fractured. Now it is quite unnecessary to attribute this to the patella of such a patient being of a very brittle texture,—an explanation that has been offered. This is exceedingly unsatisfactory, and we may explain the circumstance on another principle, namely, the lameness and inperfection of the limb consequent upon the previous injury. In all probability, when the fractured patella has united, there has been a considerable space intervening between the portions, and the interval has been filled up with ligamentous substance. This, it may be perceived, is but a poor substitute for the perfect structure of the patella: the office of the patella is nearly destroyed, for the ligamentous part plays upon the surface of the femur, nearer the centre of motion, and, consequently, the muscles lose somewhat of their lever power. The individual is, therefore, apt to trip frequently, and, consequently, he is very often called upon to make those violent exertions to save himself from falling, which it has been shown are the most likely to endanger the patella. It is thus he incurs the risk of rupturing the patella of the sound limb from having already had a fracture of the other. It is not improbable, however, that there may also be some peculiarity in the form of the projecting part of the patella, which may render its fracture more likely to occur in some persons rather than in others.*

* We have heard Mr. Bell state, in other courses of his lectures, a

The next question presented for our consideration is, What is to be done? Is the bone to be set, and the knee bandaged? There will be many occasions for our remarking the impropriety in all kinds of fracture of bandaging the limb immediately after the accident has occurred; we must delay until the attending inflammation has been subdued. In reference to the accident we are now considering, this is a part of the subject of high importance, and one which ought to be well understood. Some years ago it happened thus:—there were four cases of fractured patella admitted into the hospital, nearly all at the same time, or in quick succession, and great satisfaction was derived from the success which attended them; but at last a man was brought who had fallen from a height, and shattered the patella by striking his knee against a projecting point. The young gentleman who had the charge of all these cases, being satisfied with the practice which had proved so successful in the former instances, thought that he would do right in pursuing the same treatment in this one also. Seeing the good effects of bandaging, and not having sufficiently studied the difference of the case, he applied a bandage the very day that the patient was admitted into the hospital. The effects were but too obvious on the first view of the surgeons; the pain, swelling, and inflammation, were excessive, and—not to dwell on an unfortunate occurrence—not the limb only but the life was lost.

I am satisfied that you will make the proper use of this case. Although it happened in this hospital, yet I am bound to relate it for your instruction; it conveys an impressive lesson, and should make us consider well the difference in the nature of the various injuries by which the patella may be fractured. In a case of this kind, when there has been a violent blow upon the knee-joint, inflammation will continue for a much longer time than when the bone has been simply broken by the action of the muscles. In the case of David Keith, it is mentioned that an attempt was made to bandage the limb, but it

---

case which proves how cautious patients ought to be after the patella has been fractured. A patient was dismissed from an hospital with the portions of the patella united by a long ligament. Whilst he was carrying a burden on his head, he slipped, and the joint yielding, the whole anterior part of the knee was rent open and its cavity exposed, so that it was necessary to amputate the limb. He used to state this case as a reason why, on dismissing patients from the hospital, he ordered a splint to be put behind the knee-joint. It also affords a reason for endeavoring to bring the portions of bone as close as possible; when they are far apart, the integument is consolidated with the ligamentous substance, it loses thereby its elasticity, and the above accident may take place. Mr. Bell has suggested in these cases of fractured patella the use of the seton. When the cure has produced so far as to close the joint by a newly-formed membrane between the broken bones, he conceives it practicable to pass a seton between them, without penetrating the joint. If at the same time the portions of bone be pushed into close contact, there may be produced union, either by ossific matter, or by a short and condensed ligament.

had to be desisted from, on account of the patients complaining of pain. But let me repeat, when the patella is broken by a direct blow there is not the same separation of the portions of the patella, which occurs in the common cases; the blow breaks the bone, but still the strong fascia which covers the knee-joint,and isfastened to the patella, is entire, and prevents the splinters from being detached far from each other. In one case the patella was fractured into five different portions, yet they were all agglutinated and supported by masses of coagulable lymph, which in some parts had become ossified; this shows that there need be no anxiety about applying the bandages too early.

Bleeding and cold evaporating lotions, with all the antiphlogistic means, are first to be used, to subdue the inflammation. After this the bandages and pads may be applied so as to approximate and give support to the pieces of bone. Care is to be taken during the whole treatment to keep the leg in an extended posture: in the first place, to prevent a wider separation of the portions of the patella before the proper bandages can be applied, and because we cannot expect to bring the detached pieces close together, unless the muscles, which have a tendency to draw up the patella, are relaxed. The patient's body must be raised in bed, and he must have a bed-chair to make him sit up; or, if he grows weary of this position, attention must be paid to elevating the extended limb whenever he lies down, raising the heel by pillows, for the purpose of causing the knee to approach the pelvis, that the rectus extensor cruris may be relaxed.

It has been said that we should not try to unite this fracture by bone, and the fact that ligament is commonly the bond of union in fractures of the patella, has been regarded as a beautiful provision of nature for protecting the joint. If ossific union took place, then the patella, it has been argued, would present a rough, unpolished surface to the smooth articular cartilage of the joint, and might thus injure its delicate structure. But we need have no dread of bony union producing this mischief. I have examined, after death, four cases of fracture of the patella, consequent on direct blows received upon that bone, in which were exhibited the several stages of ossific union. They each proved that the roughness existed only on the outer surface of the bone. The specimen, which is now brought for your inspection, exhibits the pieces of bone attached closely together; and still they present an uniform surface internally, while externally, they are surrounded with masses of coagulable lymph, and of bony deposition, just as in fracture of the long bones. It is, therefore, the rule of parctice in all cases, to bring the broken portions exactly in apposition:—in the one case, with the hope of attaining ossific union from the bones being placed closely in contact with each other, or if we do not succeed in that, at least we may obtain a very short interval occupied with ligament; on the other hand, if it has been a case of fracture produced by a kick or a blow on the knee, then we may probably have success, similar to that in the case of Keith, in which we find the bone is compact, and the motion of the joint free. Several other patients have been dismissed from this hospital, in whom there has been the same reason to conclude that the union by bone had taken place.

There is a question at present much agitated among the senior members of the profession.—How comes it that the fractured patella is so seldom united by bone, and so frequently through the medium of ligament? In explanation of this we are led to consider what occurs when one of the long bones is fractured. If the humerous or femur, for example, be broken, it implies that there is great injury committed to the whole mass of flesh that surrounds the bone: it is bruised, and extravasation of blood is the consequence: the fractured ends of the bone also lacerate the surrounding textures; the blood is in this way permitted to lodge close upon the surface of the bone. Then tumefaction of the whole limb takes place,—inflammation is set up, and the blood, which was effused, undergoes a change in process of time, and coagulable lymph is substituted in its place; consolidation of the surrounding cellular membrane and periosteum proceeds gradually to make the limb firmer: there is adhesion between the two fractured ends, by means of this condensed mass, which is found fixed to the bones: this coagulable lymph gradually is converted, first into a substance of a cartilaginous hardness, and finally into true bone; spicula of bone having grown within it, they coalesce, so as to produce solid union. It appears that this great and extensive injury, this effusion of blood through the cellular membrane, this tearing of the flesh, and the consequent swelling and inflammation of the limb, all of which we might consider as disastrous, are essential for the reunion of broken bones. When we next contemplate what takes place during the fracture of the patella, produced by the action of the muscles, we perceive that there is a great difference in the circumstances. The bone has been snapped across without any corresponding injury being inflicted on the soft textures; the degree of violence is not considerable, the tumefaction that ensues is seldom very great; there is scarcely any ecchymosis. Thus there appears not to be sufficient injury inflicted for producing union by bone. We may observe the same thing when the olecranon has been fractured; or when the neck of the femur has been broken within the capsule. This last mentioned accident has given rise, it is well known, to much discussion among the most eminent of our profession. An explanation of the fact is sought, why in fractures of the neck of the femur, the union is commonly by ligament instead of by bone. It is not altogether from the difficulty of keeping the parts in apposition, that this is to be explained. In cases which I have examined after death, where the bone was fractured within the capsule, I have discovered that a thin membrane alone intervened between the portions, showing how nearly the parts had been kept approximated, yet, after all, they were not united by bone. Some other method must be used to account for the fact. The same reasoning which has just been applied to explain the want of ossific union in cases of fractured patella, may be employed here also. If the neck of the femur be broken across at its thin cervix, which is enclosed in the tough capsular ligament, the extent of the injury is very limited. There may be, perhaps, an increased secretion of the synovial fluid of the joint, but there are no cellular connexions torn up, into which the blood may be effused, and by which the broken ends of the bone may be surrounded, as in other cases; the fractured extremities are not supported by the consideration of the neighbouring parts, nor sustained by the same mass of inflamed structure, which is always found when the long bones are fractured at their centre. This appears to afford an explanation of the extreme rarity of union by bone in the case of fracture of the neck of the femur. A certain degree of violence seems essential for producing ossific union; and the neck of the thigh-bone included in its natural capsule is protected from receiving that degree of injury. A difference in the circumstances is attended with a difference in the results; for when the fracture occurs externally to the capsular ligament, then union by bone takes place. The splitting of the bone is attended with laceration of the neighbouring parts; blood is effused, and inflammation, with condensation of the surrounding muscles, are the consequences. To this point we shall recur when an opportunity again presents itself.

